# Irritant Inhalation Evokes P Wave Morphological Changes in Spontaneously Hypertensive Rats via Reflex Modulation of the Autonomic Nervous System

**DOI:** 10.3389/fphys.2021.642299

**Published:** 2021-07-27

**Authors:** J. Shane Hooper, Thomas E. Taylor-Clark

**Affiliations:** Molecular Pharmacology and Physiology, Morsani College of Medicine, University of South Florida, Tampa, FL, United States

**Keywords:** autonomic (vegetative) nervous system, TRPA1, irritant, ECG, P wave, ectopic beat, atrial fibrillation, hypertension

## Abstract

Irritant inhalation is associated with increased incidence of atrial fibrillation (AF) and stroke. Irritant inhalation acutely regulates cardiac function via autonomic reflexes. Increases in parasympathetic and sympathetic reflexes may increase atrial susceptibility to ectopic activity and the initiation of arrhythmia such as AF. Both age and hypertension are risk factors for AF. We have shown that irritant-evoked pulmonary–cardiac reflexes are remodeled in spontaneously hypertensive (SH) rats to include a sympathetic component in addition to the parasympathetic reflex observed in normotensive Wistar-Kyoto (WKY) rats. Here, we analyzed P wave morphology in 15-week old WKY and SH rats during inhalation of the transient receptor potential ankyrin 1 agonist allyl isothiocyanate (AITC). P Wave morphology was normal during vehicle inhalation but was variably modulated by AITC. AITC increased RR intervals (RRi), PR intervals, and the P Wave duration. In SH rats only, AITC inhalation increased the occurrence of negative P waves. The incidence of AITC-evoked negative P waves in SH rats was dependent on RRi, increasing during bradycardic and tachycardic cardiac cycles. Inhibition of both parasympathetic (using atropine) and sympathetic (using atenolol) components of the pulmonary–cardiac reflex decreased the incidence of negative P waves. Lastly, the probability of evoking a negative P Wave was increased by the occurrence of preceding negative P waves. We conclude that the remodeled irritant-evoked pulmonary–cardiac reflex in SH rats provides a substrate for altered P Wave morphologies. These are likely ectopic atrial beats that could provide a trigger for AF initiation in structurally remodeled atria.

## Introduction

Atrial conduction abnormalities may be caused by structural changes in the heart (e.g., hypertrophy, dilation, fibrosis) and by electrophysiological remodeling (e.g., ion channels, connexins) within cardiomyocytes (Nattel et al., 2000). Aberrant atrial conduction, particularly in the form of atrial fibrillation (AF) and atrial flutter, is a common cause of atrial dysfunction accompanied by debilitating cardiac symptoms and an increased chance of developing arterial blood clots (resulting in stroke and pulmonary embolism) (Hald et al., 2018). There are multiple risk factors for the development of AF, including advanced age, hypertension, congestive heart failure, diabetes, and obesity (Benjamin et al., 1998). Although substantial work has investigated the tendency for episodes of AF to cause electrical or structural remodeling that promote the maintenance of AF (“AF begets AF”) (Nattel et al., 2000), less is known about the direct trigger for the initiation of AF in clinical populations (Haïssaguerre et al., 1998; Hnatkova et al., 1998; O’Donnell et al., 2003; Rajawat et al., 2004; Ohkubo et al., 2008). Studies suggest that modulation of atrial conduction by the autonomic nervous system alters AF susceptibility in animal models (Shen and Zipes, 2014). Activation of both parasympathetic cholinergic pathways and sympathetic adrenergic pathways increases AF susceptibility, and simultaneous activation may be additive (Liu and Nattel, 1997; Oliveira et al., 2011; Shen et al., 2011).

Epidemiological studies have routinely shown that inhalation of air pollution, such as particulate matter (PM), is associated with cardiopulmonary morbidity and mortality, particularly in individuals with preexisting cardiovascular disease (CVD) (Pope et al., 2004; Brook et al., 2010). PM inhalation is also associated with increased incidence of stroke (Huang et al., 2019). Furthermore, in a controlled exposure study, PM inhalation increased the incidence of AF in an aged population with structural heart disease (Link et al., 2013). Inhalation of irritants and pollutants, including PM, causes changes in the autonomic control of heart rate and blood pressure, via reflexes initiated by sensory nerves innervating the airways (Taylor-Clark, 2020). Many of these irritants and pollutants activate a subset of nociceptive vagal sensory C-fibers via the gating of the transient receptor potential ankyrin 1 (TRPA1) ion channel (Bautista et al., 2005; Taylor-Clark et al., 2009; Taylor-Clark and Undem, 2010; Deering-Rice et al., 2011, 2019). In healthy animals, activation of airway vagal C-fibers causes a central reflex increase in parasympathetic drive to the heart, resulting in atropine-sensitive reflex bradycardia and hypotension (Coleridge and Coleridge, 1984; Hooper et al., 2016). Similarly, in healthy individuals, inhalation of PM evokes bradycardia (Devlin et al., 2003; Routledge et al., 2006; Peretz et al., 2008). Interestingly, PM is associated with tachycardia and hypertension in a number of clinical cohorts with preexisting CVD, including hypertension and a history of myocardial dysfunction (Peters et al., 2000; Devlin et al., 2003; Gold et al., 2005; Park et al., 2005; Chahine et al., 2007; Chuang et al., 2007). This suggests that PM-evoked modulation of cardiovascular function is remodeled by preexisting CVD. We recently showed evidence of a similar remodeling of irritant-evoked reflexes in spontaneously hypertensive (SH) rats: inhalation of irritants, including allyl isothiocyanate (AITC), the TRPA1 agonist, caused a complex brady-tachycardia accompanied by premature ventricular contractions (PVCs) in SH rats, but only bradycardia with no PVCs in the normotensive Wistar-Kyoto (WKY) rat (Hooper et al., 2019). The AITC-evoked tachycardic episodes and PVCs were blocked by β1 adrenoceptor inhibitor atenolol but not by atropine, indicating chronic hypertension remodeled irritant-evoked pulmonary–cardiac reflexes to include a *de novo* sympathetic component.

Given that (1) AF risk factors include preexisting CVD, (2) AF susceptibility is sensitive to autonomic balance, (3) inhalation of pollutants is associated with AF and stroke in susceptible individuals with preexisting CVD, and (4) preexisting CVD remodels irritant-evoked pulmonary–cardiac reflexes, we hypothesize that inhalation of AITC would cause greater atrial conduction abnormalities in SH rats compared to WKY rats. We have reanalyzed ECG data from our previous publication (Hooper et al., 2019) to study P Wave parameters. Here, we show that AITC inhalation increases the incidence of negative P waves in SH rats compared to WKY rats, and that this is dependent on the reflex activation of both parasympathetic and sympathetic drive to the heart. Negative P waves are likely ectopic atrial beats (Waldo et al., 1975), and ectopic atrial activity is considered a major trigger of paroxysmal AF (Haïssaguerre et al., 1998; O’Donnell et al., 2003; Rajawat et al., 2004; Ohkubo et al., 2008). Thus the remodeled irritant-evoked pulmonary–cardiac reflex in a model of chronic hypertension provides an electrical substrate that may be an additional risk factor in the initiation and maintenance of AF in susceptible populations.

## Materials and Methods

### ECG Acquisition and Agonist Exposure

All animal studies were approved by the University of South Florida Institutional Animal Care and Use Committee (AAALAC #000434). This data is a reanalysis of experimental studies previously described (Hooper et al., 2019). Briefly, 15-week-old male SH and WKY rats, purchased from Charles River, were implanted with a radiotelemetric device [4ET, Data Sciences International (DSI)] through a midline incision in the abdomen under controlled anesthetic (1–5% isoflurane). A trocar was then used to tunnel through the right pectoral muscle layers allowing for the negative ECG lead to be fed rostrally through the trocar and secured by a single polyethylene suture. The positive ECG lead was implanted into the lower left flank using the same procedure giving the lead II ECG position. A total of 7–10 days following surgery, ECGs were recorded from freely moving rats contained in a plexiglass chamber (4.5 × 11s″) placed on top of the DSI receiver (RPC-1). The receiver was connected to a computer running Ponemah software via an A/D converter.

Electrocardiogram were recorded continuously for 30 min during sequential exposures to ambient air, nebulized vehicle [4% ethanol in phosphate buffered saline (PBS)] and AITC (4.3 mg/ml) made up in 4% ethanol and PBS (10 min each). Exposures were performed using a Trek S (PARI Respiratory Equipment) nebulizer (4 L/min), which produces 1–5 μm particles. In some cases, rats were pretreated with either the muscarinic inhibitor atropine (1 mg/kg, i.p.) or the β1 adrenoceptor inhibitor atenolol (0.5 mg/kg, i.p.) 1 h prior to the nebulized exposures. The half-life for atropine in the rat is between 45 and 100 min (Harrison et al., 1974). As such we expect little attenuation of atropine’s effect after 60 min, consistent with the complete abolishment of AITC-evoked reflex-mediated bradycardia in Sprague Dawley rats (Hooper et al., 2016). The half-life for atenolol in the rat is 24–35 h (Tabacova and Kimmel, 2002). We expect little decrease in atenolol’s effect following 1 h. In total 15 WKY rats (9 control, 6 treated with atropine) and 25 SH rats (12 control, 9 treated with atropine, 4 treated with atenolol) were used in this study. All experiments were performed at the same time of day (0900–1100) to minimize physiological variation due to circadian rhythms.

### Data Analysis

Data was recorded at 5000 Hz and the cardiac cycle, including P, QRS, and T waves, were resolved using the Ponemah P3Plus software. In particular the beginning and end of the P Wave (Pstart and Pend) were determined. The RR interval (RRi, in ms), PR interval (PRi, in ms, defined as the interval between the Pstart and R wave), P Wave duration (Pwidth, in ms, defined as the interval between the Pstart and Pend) data were analyzed. The P Wave amplitude/height (P-H, in mV) was calculated as the amplitude of the highest or lowest point during the P Wave from the Iso-electric line at Pstart. The P-H for a biphasic P Wave was calculated as the greater of the positive and negative amplitudes.

As movement can cause interference in the ECG signal leading to software P Wave misidentification, all cycles were individually assessed to ensure proper marking and any cycle with an indiscernible P Wave was excluded from the analysis. Based upon our need for correlating P Wave parameters with heart rate on a beat-to-beat basis, we have chosen to present heart rate in the form of the RRi parameter (Heart rate = (1000/RRi) × 60). Overall mean ± SEM ECG parameter data for each condition represents the mean of all measurable events within the first 4 min of exposure for each rat. Only the first 4 min of AITC exposure were analyzed because previous studies had identified some tachyphylaxis of the reflex modulation of the cardiac cycle (Hooper et al., 2016, 2019). The raw RRi and PRi data was previously published (Hooper et al., 2019). The AITC-evoked effect on each ECG parameter was calculated for each rat by subtracting the raw parameter during vehicle inhalation from the raw parameter during AITC inhalation. The probability of evoking a negative P Wave was calculated by dividing the number of cardiac cycles with a negative P Wave by the total number of cardiac cycles. The percentage of tachycardic and bradycardic cardiac cycles in a given exposure was calculated as the percentage of RRi that were <0.875 (tachycardic) and >1.125 (bradycardic) of the average RRi taken from the first 2 min of the vehicle exposure for each animal.

In some groups (control WKY rats, control SH rats, and rats pretreated with atenolol), the mean number of negative P waves in each consecutive “train” of negative P waves was calculated for each animal. A train was defined as a cluster of consecutive cardiac cycles with negative P waves. Thus an individual negative P Wave immediately preceded and followed by positive P waves was allocated a value of 1, and consecutive negative P waves were allocated the value of the sum of the total number of negative P waves occurring before a positive P Wave occurred. As such each train had ≥1 negative P waves. An average of this data produced the mean consecutive negative P Wave data for a given challenge of a single animal. To calculate the probability of a negative P Wave occurring based upon the number of preceding consecutive negative P waves, all of the P waves from a given challenge of a single animal were classified into either success or failure for a series of escalating criteria: (1) the occurrence of a negative P Wave occurring after a positive P Wave; then (2) the occurrence of a negative P Wave occurring after a single negative P Wave; then (3) the occurrence of a negative P Wave occurring after two consecutive negative P Wave; etc.). A sum was then taken of all successes (s) and failures (f) occurring during a given challenge for each criterion across all animals in the group. The probability was then calculated using the formula: s/(s + f).

### Statistics

Data were compiled and analyzed using GraphPad software. Raw data of RRi, PRi, Pwidth, and P-H were found to have Gaussian distributions (Shapiro–Wilk test, *p* > 0.05), and were compared using ANOVA with Sidak’s multiple comparisons. The AITC-evoked effect on RRi, PRi, Pwidth, and P-H were compared between WKY and SH rats using unpaired *T*-tests. Raw data of the % of cardiac cycles that were defined as bradycardic, tachycardic, or associated with a negative P Wave were compared with Kruskal–Wallis ANOVA with Dunn’s multiple comparisons. The AITC-evoked effect on the % of cardiac cycles that were associated with a negative P Wave were compared between WKY and SH rats using the unpaired Mann–Whitney test. The Pearson correlation coefficient, *r*, was determined for the correlation between P Wave parameters (PRi, Pwidth, and P-H) and RRi. In all cases, *p* < 0.05 was considered significant.

### Chemicals

Allyl isothiocyanate, atropine (free base), and atenolol were purchased from Sigma.

## Results

**FIGURE 1:**
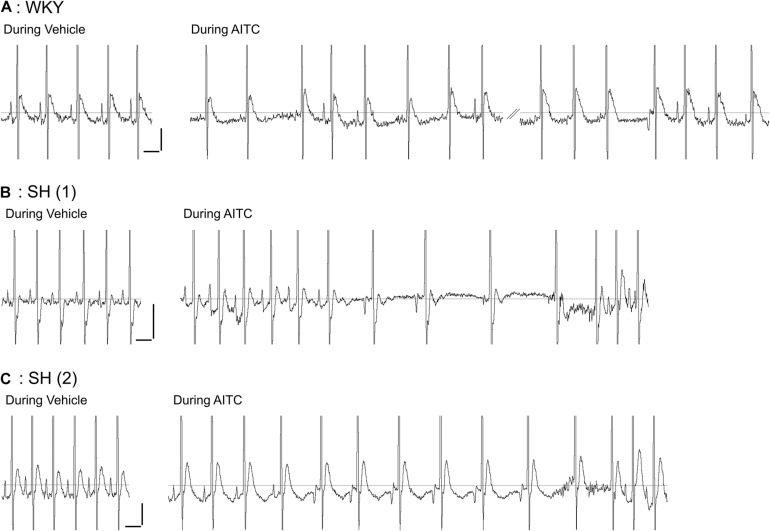
Effect of allyl isothiocyanate (AITC) inhalation on ECG in conscious Wistar-Kyoto (WKY) and spontaneously hypertensive (SH) rats. Representative ECG for a single WKY rat **(A)** and two separate SH rats **(B,C)** during exposure to vehicle (4% ethanol) and AITC (4.3 mg/ml). Note the prominent and reproducible P Wave prior to the QRS complex in all three animals during vehicle inhalation. The trace during AITC inhalation by the WKY rat **(A)** was interrupted by a period of poor ECG recording (not shown). Horizontal scale bars denote 100 ms, vertical scale bars denote 0.1 mV.

**FIGURE 2:**
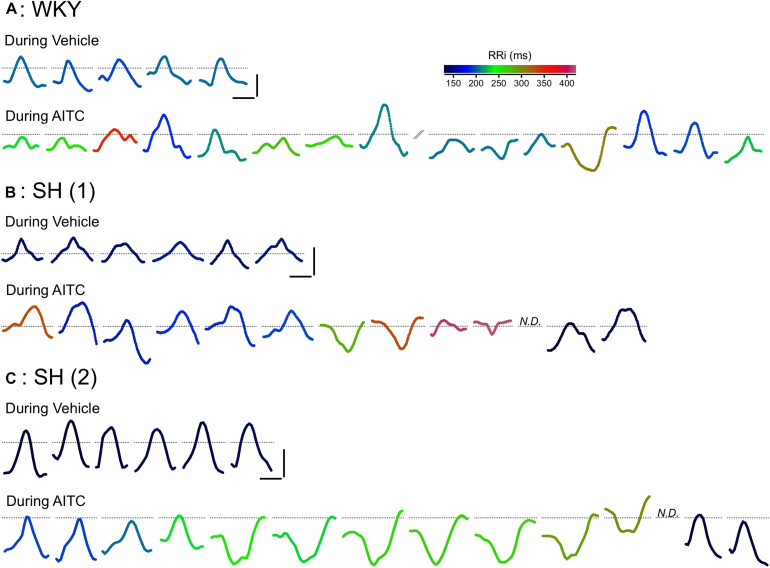
Effect of AITC inhalation on P Wave morphology in conscious WKY and SH rats. Representative consecutive P waves for vehicle (4% ethanol) and AITC (4.3 mg/ml) inhalation shown in Figure 1: a single WKY rat **(A)** and two separate SH rats **(B,C)**. The consecutive P waves shown for AITC inhalation by the WKY rat **(A)** was interrupted by a period of poor ECG recording (not shown). In some cases, the P Wave could not be discerned (N.D.). Each P Wave is colored by a rainbow color range denoting the particular RRi of that cardiac cycle. Horizontal scale bars denote 10 ms, vertical scale bars denote 0.05 mV, dotted lines denote the location of 0 mV.

**FIGURE 3:**
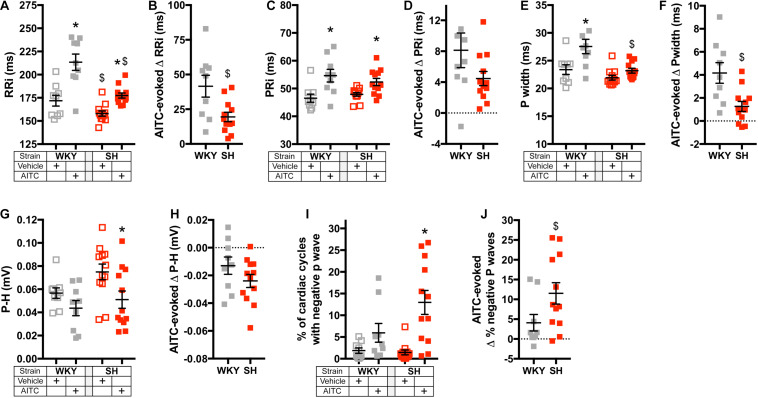
Effect of AITC inhalation on heart rate and P Wave parameters in conscious WKY and SH rats. **(A,C,E,G,I)** Mean ± SEM of RRi **(A)**, PRi **(C)**, Pwidth **(E)**, P-H **(G)**, and the % of cardiac cycles with a negative P Wave **(I)** during inhalation of vehicle (open squares) and AITC (4.3 mg/ml, closed squares) in WKY rats (gray, *n* = 9) and SH rats (red, *n* = 12). The mean ± SEM of RRi **(A)** and PRi **(C)** were previously published (Hooper et al., 2019). **(B,D,F,H,J)** Mean ± SEM of the difference between vehicle- and AITC-evoked RRi **(B)**, PRi **(D)**, Pwidth **(F)**, P-H **(H),** and the % of cardiac cycles with a negative P Wave **(J)** in WKY rats (gray, *n* = 9) and SH rats (red, *n* = 12). In **(A,C,E,G)**, * denotes significant effect of AITC compared to vehicle (*p* < 0.05) and $ denotes significant difference between WKY and SH rats (*p* < 0.05), both assessed by ANOVA with Sidak’s multiple comparisons. In **(B,D,F,H)**, $ denotes significant difference between WKY and SH rats (*p* < 0.05, unpaired *T*-test). In **(I)**, * denotes significant effect of AITC compared to vehicle (*p* < 0.05, Kruskal–Wallis ANOVA with Dunn’s multiple comparisons). In **(J)**, $ denotes significant difference between WKY and SH rats (*p* < 0.05, unpaired Mann–Whitney test).

We had previously investigated the effect of inhalation of nebulized vehicle (4% ethanol in PBS) and AITC (4.3 mg/ml) on the cardiac cycle in conscious WKY and SH rats using electrocardiogram (ECG) radiotelemetry (Hooper et al., 2019). Here, we reanalyzed the data to explore in detail the effect of AITC inhalation on P Wave characteristics (Figures 1–3), as these can be indicators of atrial susceptibility to clinically relevant arrhythmia. During the inhalation of vehicle, the cardiac cycle and P Wave morphology were relatively stable (Figures 1, 2). While the RRi of SH rats was significantly shorter than WKY rats during vehicle inhalation (*p* < 0.05), there were no significant differences in PRi, Pwidth, and P-H between the strains (*p* > 0.05) (Figures 3A,C,E,G). Inhalation of AITC caused appreciable changes in the cardiac cycle and P Wave morphology (Figures 1, 2). In WKY rats, AITC induced a significant increase in RRi compared to vehicle (*p* < 0.05, Figure 3A). This AITC-evoked bradycardia was accompanied with an increase in PRi and Pwidth (*p* < 0.05, Figures 3C,E), although there was no significant effect on the P-H (*p* > 0.05, Figure 3G). In SH rats, AITC significantly increased RRi (*p* < 0.05, Figure 3A), although this effect was less than that observed in WKY rats (*p* < 0.05, Figure 3B). AITC also increased PRi (*p* < 0.05, Figure 3C) in SH rats, but had no effect on Pwidth (*p* > 0.05, Figure 3E). In contrast to WKY, AITC caused a significant decrease in P-H in SH rats (*p* < 0.05, Figure 3G). The substantial effect of AITC inhalation on the RRi was also reflected in changes in P Wave morphology: converting the simple monophasic positive P waves observed during vehicle inhalation into notched, biphasic, and even inverted (i.e., negative) P waves in some cardiac cycles during AITC inhalation (Figures 1, 2). In particular, we found that AITC caused an increase in the % of cardiac cycles with negative P waves in SH rats (*p* < 0.05) but not in WKY rats (*p* > 0.05) (Figures 3I,J).

**FIGURE 4:**
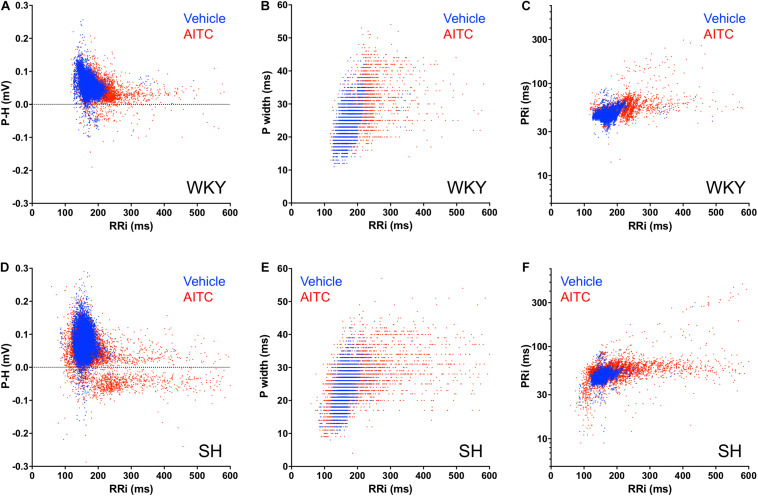
Correlation of RRi with P Wave parameters for individual cardiac cycles during AITC and vehicle inhalation. Each cardiac cycle is represented by either a blue dot (during vehicle inhalation) or a red dot (during AITC inhalation). **(A,D)** Correlation of RRi with P-H. **(B,E)** Correlation of RRi with P width. **(C,F)** Correlation of RRi with PRi. **(A–C)** Events in WKY rats (*n* = 9 animals, 8220 cycles during vehicle, 4224 cycles during AITC). **(D–F)** Events in SH rats (*n* = 12 animals, 11,745 cycles during vehicle, 9558 cycles during AITC).

Given the disordered effect of AITC inhalation on RRi and P Wave morphology, we investigated the correlation of P Wave characteristics with RRi on a beat-to-beat basis. During AITC exposure, many RRi in WKY rats were substantial prolonged (i.e., bradycardic cardiac cycles) compared to vehicle (Figure 4A). Increased RRi in WKY rats was negatively correlated with P-H (*p* < 0.05, *r*^2^ = 0.11), but few P-H values were actually negative (i.e., inverted). The AITC-evoked effect on RRi in WKY rats positively correlated with increases in Pwidth (*p* < 0.05, *r*^2^ = 0.27, Figure 4B) and PRi (*p* < 0.05, *r*^2^ = 0.23, Figure 4C). As seen previously in SH rats (Hooper et al., 2019), the distribution of RRi during AITC inhalation was complex: AITC increased the occurrence of both prolonged RRi (i.e., bradycardic cardiac cycles) and shortened RRi (i.e., tachycardic cardiac cycles) compared to vehicle (Figure 4D). Interestingly, AITC evoked negative P waves across the entire spectrum of RRi in the SH rats. Similar to WKY, the AITC-evoked effect on RRi in SH rats was positively correlated with Pwidth (*p* < 0.05, *r*^2^ = 0.29, Figure 4E) and PRi (*p* < 0.05, *r*^2^ = 0.16, Figure 4F).

**FIGURE 5:**
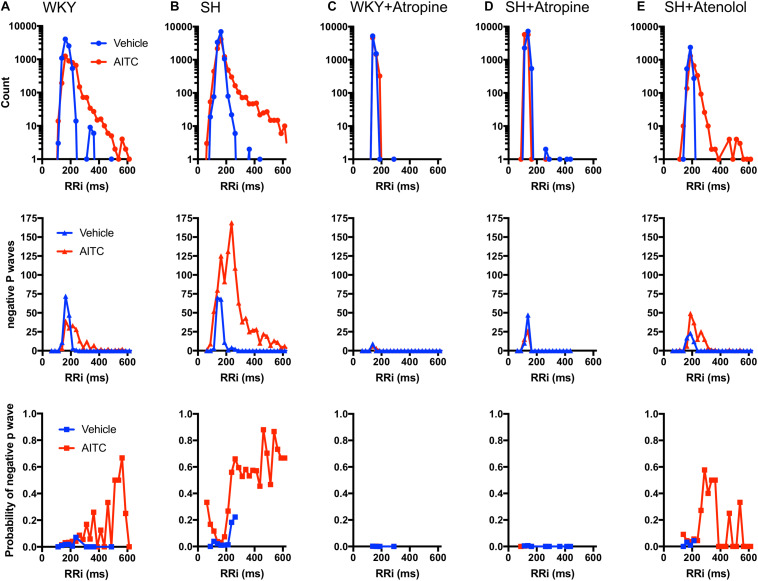
Correlation of RRi with incidence of negative P waves during AITC and vehicle inhalation. Each cardiac cycle from WKY rats (**A**, *n* = 9 animals, 8220 cycles during vehicle, 4224 cycles during AITC), SH rats (**B**, *n* = 12 animals, 11,745 cycles during vehicle, 9558 cycles during AITC), WKY rats pretreated with 1 mg/kg atropine **(C**, 6 animals, 6771 cycles during vehicle, 6363 cycles during AITC), SH rats pretreated with 1 mg/kg atropine (**D**, 9 animals, 10,104 cycles during vehicle, 11,511 cycles during AITC), and SH rats pretreated with 0.5 mg/kg atenolol **(E**, *n* = 4 animals, 3185 cycles during vehicle, 2601 cycles during AITC) was grouped by RRi into 25 ms bins. For each RRi bin during vehicle (blue) and AITC (red) inhalation the number of cardiac cycles recorded (top), the number of cycles with negative P waves (middle) and the probability of a given cycle having a negative P Wave (bottom) is shown.

In order to compare the incidence of negative P waves at different RRi we generated histograms of the number of cardiac cycles, the number of negative P waves and the probability of a P Wave being negative in relation to RRi. Furthermore, we added datasets from WKY and SH rats pretreated with atropine (1 mg/kg) and SH rats pretreated with atenolol (0.5 mg/kg), in order to determine the contribution of muscarinic receptors (i.e., parasympathetic activity) and β1 receptors (i.e., sympathetic activity), respectively, to the generation of negative P waves during vehicle and AITC inhalation. In WKY rats, few negative P waves were evoked during vehicle (*n* = 134) and, given the substantial number of cardiac cycles recorded (*n* = 8220) this indicated that vehicle has a very low probability of evoking a negative P Wave at any RRi (Figure 5A). During inhalation of AITC by WKY rats, the probability of evoking a negative P Wave appeared to increase for prolonged RRi (i.e., bradycardic cardiac cycles) (Figure 5A). In SH rats, few negative P waves were evoked during vehicle and so in general there was a very low probability of evoking a negative P Wave – although this probability appeared to increase with the few RRi > 220 ms (Figure 5B). Interestingly, the probability of evoking a negative P Wave in SH rats exposed to AITC was highly dependent on the individual RRi – the probability was very low at “normal” RRi but increased robustly at RRi<120 and >220 ms. These data suggest that atrial conduction at resting RRi is normal in SH rats, but it is disturbed for RRi outside of the resting range. As shown previously (Hooper et al., 2019), atropine abolished AITC-evoked bradycardia in both WKY and SH, consistent with the established role of vagal parasympathetic signaling in irritant-evoked pulmonary–cardiac reflexes. Consequently, no negative P waves were observed during RRi >220 ms (Figures 5C,D). Previously, we had shown that atenolol did not prevent AITC-evoked bradycardia in SH rats (Hooper et al., 2019). Here we found that AITC-evoked RRi > 220 ms in SH rats pretreated with atenolol had a high probability of having a negative P Wave (Figure 5E). Given that atropine completely abolished AITC-evoked modulation of RRi in WKY (thus indicating the reflex was entirely mediated by parasympathetic/muscarinic signaling), there was little rationale for assessing the contribution of sympathetic signaling in mediating the AITC-evoked response in the original study (Hooper et al., 2019).

**FIGURE 6:**
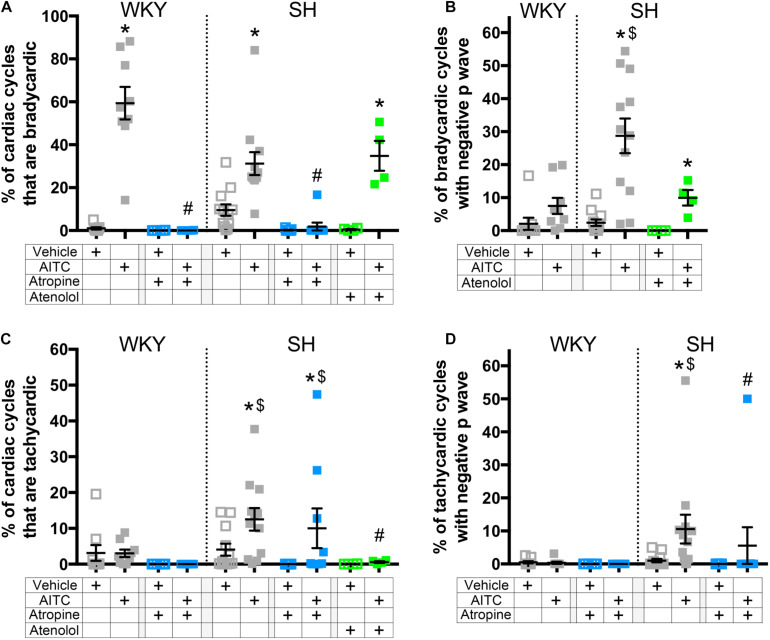
The effect of AITC inhalation on the incidence of negative P waves during bradycardia and tachycardia in conscious WKY and SH rats. **(A)** Mean ± SEM of the % of cardiac cycles which were classified as bradycardic. **(B)** Mean ± SEM of the % of bradycardic cardiac cycles with a negative P Wave. **(C)** Mean ± SEM of the % of cardiac cycles which were classified as tachycardic. **(D)** Mean ± SEM of the % of tachycardic cardiac cycles with a negative P Wave. Data was recorded during vehicle (open squares) and AITC (4.3 mg/ml, closed squares) inhalation in control WKY and SH rats (gray, *n* = 9 and 12, respectively), WKY and SH rats pretreated with 1 mg/kg atropine (blue, *n* = 6 and 9, respectively), and SH rats pretreated with 0.5 mg/kg atenolol (green, *n* = 4). The symbol * denotes significant effect of AITC compared to vehicle (*p* < 0.05), $ denotes significant difference between WKY and SH rats (*p* < 0.05), and # denotes significant effect of autonomic drugs compared to control (*p* < 0.05), each assessed in Kruskal–Wallis ANOVA with Dunn’s multiple comparisons.

Resting heart rate varies within cohorts of both WKY and SH strains (assessed at the same age) (Figure 3A). Thus it is not possible to ascribe a certain absolute value of RRi as a threshold for either bradycardia or tachycardia. We therefore defined thresholds for bradycardic cardiac cycles and tachycardic cardiac cycles for each rat (see methods) and then calculated the probability of evoking a negative P Wave in these 2 categories (Figure 6). AITC caused a significant increase in the % of cardiac cycles that were bradycardic in control WKY and control SH rats (*p* < 0.05, Figure 6A). AITC-evoked bradycardic cardiac cycles were abolished by atropine in both WKY and SH rats (*p* < 0.05, Figure 6A). AITC also caused an increase in the % of cardiac cycles that were bradycardic in SH rats treated with atenolol (*p* < 0.05, Figure 6A), which was not significantly different to control SH rats (*p* > 0.05). Importantly, AITC robustly increased the % of bradycardic cardiac cycles that had negative P waves in control SH rats (*p* < 0.05, Figure 6B), but not in control WKY rats (*p* > 0.05). In addition, AITC increased the % of bradycardic cardiac cycles that had negative P waves in SH rats treated with atenolol (*p* < 0.05, Figure 6B). This AITC-evoked response in SH rats treated with atenolol appeared to be smaller in magnitude compared to control SH rats, but this did not reach significance (*p* > 0.05). The % of bradycardic beats that had negative P waves in the atropine-treated groups could not be calculated because atropine eliminated the bradycardic cardiac cycles.

Allyl isothiocyanate caused an increase in the % of cardiac cycles that were tachycardic in control SH rats (*p* < 0.05, Figure 6C), but not in control WKY rats. AITC-evoked tachycardia in SH rats was abolished by atenolol (*p* < 0.05), but was unaffected by atropine, indicating that it is likely dependent on reflex sympathetic nerve activation. AITC caused an increase in the % of tachycardic cardiac cycles that had negative P waves in control SH rats (*p* < 0.05, Figure 6D), but not in control WKY rats. Interestingly, AITC failed to increase the % of tachycardic cardiac cycles that had negative P waves in SH rats treated with atropine (*p* > 0.05, Figure 6D), despite the numerous tachycardic cardiac cycles evoked in these animals. The % of tachycardic beats that had negative P waves in the atenolol-treated SH rats could not be calculated because atenolol eliminated the tachycardic cardiac cycles. Overall, the data suggests that SH rats have a greater susceptibility to evoke negative P waves in cardiac cycles with “non-normal” RRi than WKY rats, and that this is sensitive to autonomic blockade of both the parasympathetic and sympathetic control of the heart.

**FIGURE 7:**
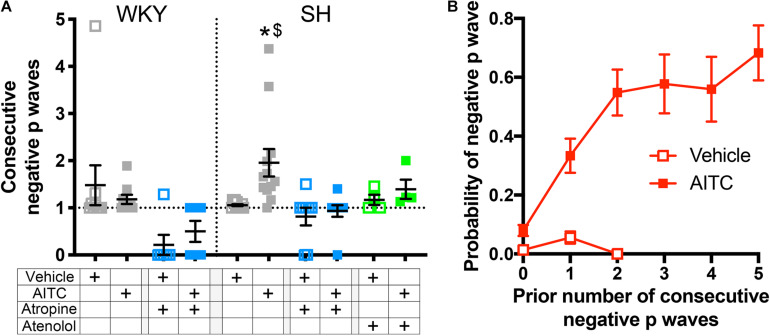
Allyl isothiocyanate inhalation increases the probability of consecutive negative P waves in SH rats. **(A)** Mean ± SEM of the average number of negative P waves in each consecutive “train” of negative P waves during vehicle (open squares) and AITC (4.3 mg/ml, closed squares) inhalation in control WKY and SH rats (gray, *n* = 9 and 12, respectively), WKY and SH rats pretreated with 1 mg/kg atropine (blue, *n* = 6 and 9, respectively), and SH rats pretreated with 0.5 mg/kg atenolol (green, *n* = 4). The symbol * denotes significant effect of AITC compared to vehicle (*p* < 0.05) and $ denotes significant difference between WKY and SH rats (*p* < 0.05), both assessed in Kruskal–Wallis ANOVA with Dunn’s multiple comparisons. **(B)** Mean ± SEM the probability of a given cycle having a negative P Wave in control SH rats (*n* = 12) during vehicle (open squares) and AITC (4.3 mg/ml, closed squares) as a function of the prior number of consecutive negative P waves.

Analysis of individual ECG records showed a tendency of negative P waves to cluster (Figures 1, 2). We calculated the mean number of negative P waves in each consecutive “train” of negative P waves. This was approximately one for all groups during vehicle inhalation – i.e., negative P waves were only found singularly – with the exception of atropine-treated WKY and SH rats which often had no negative P waves at all (Figure 7A). In control SH rats, AITC inhalation evoked a significant increase in the mean number of negative P waves in each consecutive “train” (*p* < 0.05, Figure 7A). AITC failed to increase the mean number of negative P waves in each consecutive “train” in the other groups (*p* > 0.05). Analysis of the probability that a given P Wave was negative in control SH rats during treatment with AITC showed that the probability rose from 0.08 ± 0.02 immediately following a single positive P Wave to 0.33 ± 0.06 immediately following a single negative P Wave (Figure 7B). Thus, the probability of AITC evoking a negative P Wave in control SH rats was significantly increased by the number of preceding negative P waves (*p* < 0.05, Figure 7B).

## Discussion

Airborne pollutants such as PM, ozone, diisocyanates, and acrolein trigger acute cardiopulmonary responses via the TRPA1-mediated activation of nociceptive sensory nerves innervating the airways (Bautista et al., 2005; Taylor-Clark et al., 2009; Taylor-Clark and Undem, 2010; Deering-Rice et al., 2011, 2019; Taylor-Clark, 2020). Activation of nociceptive airway sensory nerves causes a vagal–vagal central reflex-mediated bradycardia (Coleridge and Coleridge, 1984; Hooper et al., 2016). We have previously shown that this irritant-evoked pulmonary–cardiac reflex is remodeled in hypertensive rats such that, in addition to the atropine-sensitive reflex bradycardia, there is an additional *de novo* atenolol-sensitive reflex tachycardia with accompanying PVCs (Hooper et al., 2019). Here, we have found that hypertensive rats are also more susceptible to atrial conduction abnormalities evoked by nociceptive pulmonary–cardiac reflexes, and this is dependent on the activation of both parasympathetic and sympathetic signaling.

P Wave polarity and morphology is an indicator of atrial electrical conduction. If initiated at the sinoatrial node, P waves (in lead II position) are positive in direction without major notches (Waldo et al., 1975). Gross deviations from this shape suggests ectopic P Wave initiation, the source of which can be identified in 12-lead ECG studies of the human heart (Waldo et al., 1977; O’Donnell et al., 2003; Rajawat et al., 2004; Ohkubo et al., 2008; Censi et al., 2016). For example, ectopic activity originating near the pulmonary veins (in the left atria) can cause notched or negative P waves in lead II (Waldo et al., 1977; Rajawat et al., 2004; Ohkubo et al., 2008). Ectopic atrial activity is observed in all AF patients, with many unique P waves originating from the pulmonary veins (Haïssaguerre et al., 1998; O’Donnell et al., 2003; Rajawat et al., 2004; Ohkubo et al., 2008). The importance of these ectopic P waves is shown by the observation that ablation of regions surrounding the pulmonary vein can eliminate the ectopic beats and reduce AF (Haïssaguerre et al., 1998). The tendency to evoke ectopic P waves is negatively correlated with the effective refractory period (ERP) of atrial tissue, which is shortest near the pulmonary veins (Oliveira et al., 2011; Fan et al., 2019). There are multiple factors that promote aberrant atrial conduction including atrial hypertrophy and fibrosis (structural changes) and by changes in intrinsic electrical components (e.g., ion channels, connexins) within cardiomyocytes (Nattel et al., 2000). Conditions which promote these factors, such as age, congestive heart failure, hypertension, and diabetes are risk factors for AF (Benjamin et al., 1998). Furthermore, it is likely that structural changes induce electrical remodeling and vice versa. This complexity culminates in the concept that “AF begets AF” (Nattel et al., 2000), which can be seen in experimentally paced animals (Hayashi et al., 2002; Ogawa et al., 2009; Oliveira et al., 2011) and in the progression of clinical AF from paroxysmal to persistent AF in some patients (O’Donnell et al., 2003). AF is associated with a progressive decrease in ERP (Hayashi et al., 2002; Anyukhovsky et al., 2005; Fan et al., 2019), which provides a substrate for re-entry – promoting AF maintenance or further AF initiations.

Identifying the source of ectopic P waves in the less characterized rat heart with a single pair of leads (lead II) is not possible. Nevertheless, our data demonstrates a profound change in P Wave morphology in both WKY and SH rats during inhalation of AITC compared to vehicle: during vehicle inhalation, P waves are positive, and regularly shaped; whereas AITC causes variable changes in some P waves, decreasing their amplitude, increasing the PRi, introducing notches and causing inversions (negative P waves) (see Figures 1, 2). The decreased amplitude and increased PRi is consistent with the slowing of atrial conduction, likely due to the actions of acetylcholine on atrial M2 muscarinic receptors following increases in reflex parasympathetic activity (Hooper et al., 2019). Notched or negative P waves are likely ectopic atrial beats (Waldo et al., 1975), and this is consistent with heterogeneity of conduction throughout the rat atria and pacemaker activity near the rat pulmonary vein (Masani, 1986; Maupoil et al., 2007; Fan et al., 2019; Logantha et al., 2019). AITC inhalation caused an increase in negative P waves in SH rats but not WKY rats, despite the observation that AITC-evoked increases in PRi were similar between the strains and AITC-evoked increases in RRi and Pwidth were greater in the WKY rat (Figures 3, 4). The probability of a P wave being negative in SH rats was highly dependent on RRi – the probability was negligibly low at heart rates consistent with sinus rhythm but increased dramatically for bradycardic and tachycardic cardiac cycles (Figure 5).

Spontaneously hypertensive rats have decreased ERP compared to WKY rats, and there is greater heterogeneity of ERP across the atria in SH rats (Lau et al., 2013). Thus SH atria are intrinsically arrhythmogenic. However, our data suggest that in addition to this intrinsic susceptibility, the increased number of negative P waves evoked by AITC in SH rats is dependent on the aberrant remodeled pulmonary–cardiac reflex, which includes both atropine-sensitive and atenolol-sensitive components. Atropine had no effect on AITC-evoked tachycardia in the SH rat (Figure 6C), but abolished the negative P waves associated with that tachycardia (Figure 6D). Similarly, atenolol had no effect on AITC-evoked bradycardia in SH rats (Figure 6A), but prevented consecutive negative P waves from forming (Figure 7A). There was also a trend for atenolol to decrease the % of negative P waves associated with AITC-evoked bradycardia, but this failed to reach significance (Figure 6B). Overall, the data indicate that both parasympathetic and sympathetic reflexes contributed to the initiation of ectopic P waves during irritant inhalation. Despite the current observations that the AITC-evoked changes in RRi and P Wave polarity are abolished by atropine and/or atenolol in WKY and SH rats, we cannot definitely rule out a direct effect of AITC on the atrial tissue. Further inhalation studies with other nociceptor stimulants may provide clarity on this issue.

What can explain this reflex modulation of P Wave polarity? Vagal nerve stimulation causes atropine-sensitive bradycardia and alters the morphology of P waves (Ninomiya, 1966). Outside of the sinoatrial node, muscarinic signaling increases I_*KACh*_ currents, resulting in decreased action potential duration (Chen et al., 2014). Furthermore, this effect is heterogeneous (Ninomiya, 1966; Liu and Nattel, 1997), thus parasympathetic signaling reduces atrial ERP in a spatially and temporally heterogeneous manner, and consequently increases the susceptibility and duration of experimentally induced AF (Liu and Nattel, 1997; Oliveira et al., 2011). Stimulation of sympathetic signaling to the heart also decreases atrial ERP (Liu and Nattel, 1997), principally by modulating cardiomyocyte Ca^2+^ handling (Chen et al., 2014). However, this effect is not spatially heterogeneous, and thus sympathetic stimulation alone only marginally increases susceptibility to experimentally induced AF (Liu and Nattel, 1997). Nevertheless, there is evidence that coactivation of parasympathetic and sympathetic activity correlates with atrial arrhythmia and AF susceptibility (Shen et al., 2011). Furthermore, stellate ganglion ablation reduced experimentally induced AF (Ogawa et al., 2009; Shelton et al., 2018). This suggests that an increase in sympathetic signaling to the heart can also contribute to atrial conduction dysfunction. Here, we have shown that AITC inhalation in the SH rat increases the probability of negative P Wave initiation, and this appears dependent on both parasympathetic and sympathetic signaling. Although the current data is consistent with a role of autonomic reflexes in the modulation of the atrial ERP, further studies are required to directly measure the ERP in the SH heart during AITC-evoked reflex modulation.

Although AITC inhalation induced a significant increase in ectopic P waves in SH rats, we wish to emphasize that we found no evidence of AF. This may be due to the age of the rats (15 weeks) used in this study. Tachypacing-induced atrial tachyarrhythmia were significantly increased in 11 month old SH rats compared to age-mated WKY rats or 3 month old SH rats (Choisy et al., 2007), and 55 week old SH rats have spontaneous premature atrial contractions and atrial tachycardia unlike WKY rats or 14 week old SH rats (Scridon et al., 2012). Aging leads to increased cardiac fibrosis and hypertrophy in the SH rat (Choisy et al., 2007; Lau et al., 2013), which was associated with P Wave abnormalities, ventricular arrhythmia, and heart failure at >12 months (Pfeffer et al., 1976; Dunn et al., 1978). Despite the lack of AF, AITC-evoked negative P waves in SH rats were clustered in consecutive “trains,” with the probability of a negative P Wave increasing dramatically with the occurrence of preceding negative P waves. This may have occurred because the electrical conditions conducive to increased ectopic atrial activity (decreased atrial ERP with spatially and temporally heterogeneity) occurred over a period longer than an individual cardiac cycle. Such a hypothesis would be consistent with the importance of sympathetic signaling in these responses which modulates cardiac rhythms over slower cycles than the beat-to-beat modulation of parasympathetic signaling (Thoren et al., 1979; Chen et al., 2014). Nevertheless, we cannot discount the additional possibility that the occurrence of a negative P Wave itself increased the probability of a subsequent negative P Wave. This may reflect early stages of electrical and structural remodeling in the SH (Dunn et al., 1978), and is consistent with reports that, in some clinical populations, ectopic atrial beats, especially originating from the pulmonary veins (negative P Wave in lead II), can initiate AF (Haïssaguerre et al., 1998; O’Donnell et al., 2003; Rajawat et al., 2004; Ohkubo et al., 2008).

In conclusion, we have shown that the remodeled irritant-evoked pulmonary–cardiac reflex (composed of both parasympathetic and sympathetic components) in the SH rat provides an electrical substrate that induces negative P waves. These ectopic P waves could trigger AF initiation in structurally remodeled atria. Inhalation of the selective TRPA1 agonist AITC activates the same neural pathways as air pollution. Air pollution has previously been shown to increase the incidence of AF in an aged population with structural heart disease (57% of whom also had hypertension) (Link et al., 2013). Thus the remodeling of pollution-sensitive pulmonary–cardiac reflexes may be an additional risk factor in the initiation and maintenance of AF in susceptible populations.

## Data Availability Statement

The raw data supporting the conclusions of this article will be made available by the authors, without undue reservation.

## Ethics Statement

The animal study was reviewed and approved by University of South Florida Institutional Animal Care and Use Committee (AAALAC #000434).

## Author Contributions

TT-C developed the hypothesis. JH performed all the experiments and analyzed all the raw data. JH and TT-C completed the analysis and wrote the manuscript. Both authors contributed to the article and approved the submitted version.

## Conflict of Interest

The authors declare that the research was conducted in the absence of any commercial or financial relationships that could be construed as a potential conflict of interest.

## Publisher’s Note

All claims expressed in this article are solely those of the authors and do not necessarily represent those of their affiliated organizations, or those of the publisher, the editors and the reviewers. Any product that may be evaluated in this article, or claim that may be made by its manufacturer, is not guaranteed or endorsed by the publisher.
